# Using smartphone accelerometer data to obtain scientific mechanical-biological descriptors of resistance exercise training

**DOI:** 10.1371/journal.pone.0235156

**Published:** 2020-07-15

**Authors:** Claudio Viecelli, David Graf, David Aguayo, Ernst Hafen, Rudolf M. Füchslin

**Affiliations:** 1 Institute of Molecular Systems Biology, ETH Zurich, Zurich, Switzerland; 2 Institute of Applied Mathematics and Physics, Zurich University of Applied Sciences Zurich, Winterthur, Switzerland; 3 Kieser Training AG, Zürich, Switzerland; 4 European Centre for Living Technology, Ca' Bottacin, Venice, Italy; Cardiff University, UNITED KINGDOM

## Abstract

**Background:**

Single repetition, contraction-phase specific and total time-under-tension (TUT) are crucial mechano-biological descriptors associated with distinct morphological, molecular and metabolic muscular adaptations in response to exercise, rehabilitation and/or fighting sarcopenia. However, to date, no simple, reliable and valid method has been developed to measure these descriptors.

**Objective:**

In this study we aimed to test whether accelerometer data obtained from a standard smartphone placed on the weight stack can be used to extract single repetition, contraction-phase specific and total TUT.

**Methods:**

Twenty-two participants performed two sets of ten repetitions of their 60% one repetition maximum with a self-paced velocity on nine commonly used resistance exercise machines. Two identical smartphones were attached on the resistance exercise weight stacks and recorded all user-exerted accelerations. An algorithm extracted the number of repetitions, single repetition, contraction-phase specific and total TUT. All exercises were video-recorded. The TUT determined from the algorithmically-derived mechano-biological descriptors was compared with the video recordings that served as the gold standard. The agreement between the methods was examined using Limits of Agreement (LoA). The association was calculated using the Pearson correlation coefficients and interrater reliability was determined using the intraclass correlation coefficient (ICC 2.1).

**Results:**

The error rate of the algorithmic detection of single repetitions derived from two smartphones accelerometers was 0.16%. Comparing algorithmically-derived, contraction-phase specific TUT against video, showed a high degree of correlation (r>0.93) for all exercise machines. Agreement between the two methods was high on all exercise machines as follows: LoA ranged from -0.3 to 0.3 seconds for single repetition TUT (0.1% of mean TUT), from -0.6 to 0.3 seconds for concentric contraction TUT (7.1% of mean TUT), from -0.3 to 0.5 seconds for eccentric contraction TUT (4.1% of mean TUT) and from -1.9 to 1.1 seconds for total TUT (0.5% of mean TUT). Interrater reliability for single repetition, contraction-phase specific TUT was high (ICC > 0.99).

**Conclusion:**

Data from smartphone accelerometer derived resistance exercise can be used to validly and reliably extract crucial mechano-biological descriptors. Moreover, the presented multi-analytical algorithmic approach enables researchers and clinicians to reliably and validly report missing mechano-biological descriptors.

## Introduction

Skeletal muscle is one of the most important tissues of the human body. It comprises up to 40% of the body mass [1] and adapts to stimuli such as contractile activity, substrate supply, environmental factors, loading conditions and contributes to mechanical and metabolic functions [2].

Mechanically, its main function is to convert chemical into mechanical energy that can be used for force production thus enabling locomotion. Metabolically, skeletal muscle is a sink for substrates such as amino acids, carbohydrates, fatty acids, minerals and inorganic salts and it contributes to the maintenance of the basal energy metabolism. In times of starvation, it is able to maintain key tissue protein mass and plasma glucose levels [1] relatively constant, provided that skeletal muscle mass is sufficient. Therefore, skeletal muscle mass regulates metabolic homeostasis and contributes substantially to survival [3,4].

Muscle mass is lost during ageing due to age-related sarcopenia. This process is characterized by a progressive and generalized loss of skeletal muscle mass and strength. Overall, this loss negatively affects muscle strength, metabolic rate, aerobic capacity and thus, a person's functional capacity [5]. Moreover, the results of these processes associated with sarcopenia are related to increased risk of adverse outcomes such as physical disability, poor quality of life and ultimately death [5–7]. After reaching a peak in adult years, skeletal muscle mass gradually begins to decline at approximately age 45 [8–10]. The results of several longitudinal studies suggest that muscle mass declines by approximately 6% per decade after mid-life [11]. It is estimated that an individual loses 30% of individual peak muscle mass by the age of 80 [8]. Given that skeletal muscle mass accounts for up to 40% of an individual total body mass and 50–75% of all body proteins [1], the progressive loss of muscle mass has a fundamental impact on health and quality of life in the elderly population. The close link between skeletal muscle mass and bone mineral density leads to bone loss when skeletal muscle mass deteriorates [12]. Osteoporosis, the loss of bone mass [13], together with sarcopenia represent major clinical problems. The impairment of locomotory functions leads to compromised balance and increases the risk of falls promoting osteoporotic fractures [14]. Hence, low skeletal muscle mass is a driver of public medical costs because hospitalization within this cohort has a high prevalence [15]. It was estimated that healthcare costs linked to sarcopenia amounted up to 18.5 billion USD in the USA in the year 2000 [16].

It is well established that resistance exercise provides a potent anabolic stimulus to increase muscle mass [17,18] in men and women of all ages [19]. Therefore, as it combats and/or reverses sarcopenia, restores and recharges metabolism, improves adipose tissue oxidation, increases bone mineral density and prevents type 2 diabetes, resistance exercise is considered medicine [20].

However, despite receiving significant scientific attention, effective and/or efficient manipulation of resistance exercise mechano-biological descriptors inducing hypertrophy and/or strength remains unclear to date [21–25]. Although, extensively reviewed elsewhere [26], relevant mechano-biological descriptors (*e*.*g*. fractional and/or temporal distribution of contraction phases) have, for the most part, been neglected, until now. We recognize that impracticability of recording these descriptors may have contributed to this disparity.

Mobile technologies (*e*.*g*. smartphones, sensors, *etc*.) offer new possibilities for reliable, cheap and easy-to-use data acquisition that may help to optimize the outcome of resistance training efforts. Smartphones are encountered ubiquitously and are powerful portable computers, containing a plethora of accurate sensors that are already embedded in a versatile software environment. As such, smartphones can capture data from different sensors (*e*.*g*. accelerometers, gyroscope, *etc*.) and analyze them in real-time, while providing direct feedback and store data for further analysis. Compared to self-reports, sensor-captured data provide more accurate summaries of both cardiorespiratory and resistance exercise [27]. The smartphone's built-in inertial sensors (*i*.*e*. accelerometers) have proved to supply valid and reliable data during static [28] and dynamic applications [29,30]. Thus, smartphone sensors may enable high spatiotemporal resolution mapping of resistance exercise derived data.

This study aimed to examine whether smartphones can be used to I) collect real-world dynamic resistance exercise data, and II) from there derive valid and reliable contraction-specific mechano-biological descriptors (*i*.*e*. the temporal distribution of contraction phases). We hypothesized that accelerometer data of real-world dynamic resistance exercises, recorded by a smartphone placed on the weight stack, can be used to algorithmically extract contraction-specific mechano-biological descriptors.

## Material and methods

### Ethics statement

The study has been approved by the ethics committee of Swiss Federal Institute of Technology Zurich (ETH Zurich, Zurich, Switzerland) and conducted in accordance with the Declaration of Helsinki.

All participants received oral and written information about all procedures of the study and signed a written informed consent.

### Design

The study investigated whether mechano-biological descriptors, *i*.*e*. the temporal distribution of contraction modes, number of repetitions and total time-under-tension (TUT) could be extracted from accelerometer derived real-world dynamic resistance exercise data on different resistance exercise machines. Nine resistance exercise machines were selected at the gym located at ETH Zurich. The selected machines comprised the most often chosen exercises in a whole-body workout and were as follows: Adductor, Abductor, Chest Press, Leg Curl, Leg Extension, Leg Press, Lower Back, Total Abdominal and Vertical Traction (Technogym, Cesana, Italy). Video recordings, which are considered the gold standard, were made for all exercises.

### Participants

Twenty-two healthy volunteers between the ages of 19 and 70 years were recruited via academic mailing lists, flyers and word-to-mouth. All participants completed a routine health questionnaire before giving written informed consent to participate in the study. In the case of one of the volunteers who exhibited a potential health-related issue, the consent of a physician was obtained.

### Equipment

Accelerometer data were collected using two Nexus 6P (Huawei Technologies Co., Ltd., Shenzhen, China) smartphones with built-in 3-axis accelerometer BMI160 (Robert Bosch GmbH, Stuttgart, Germany).

**Fig 1:**
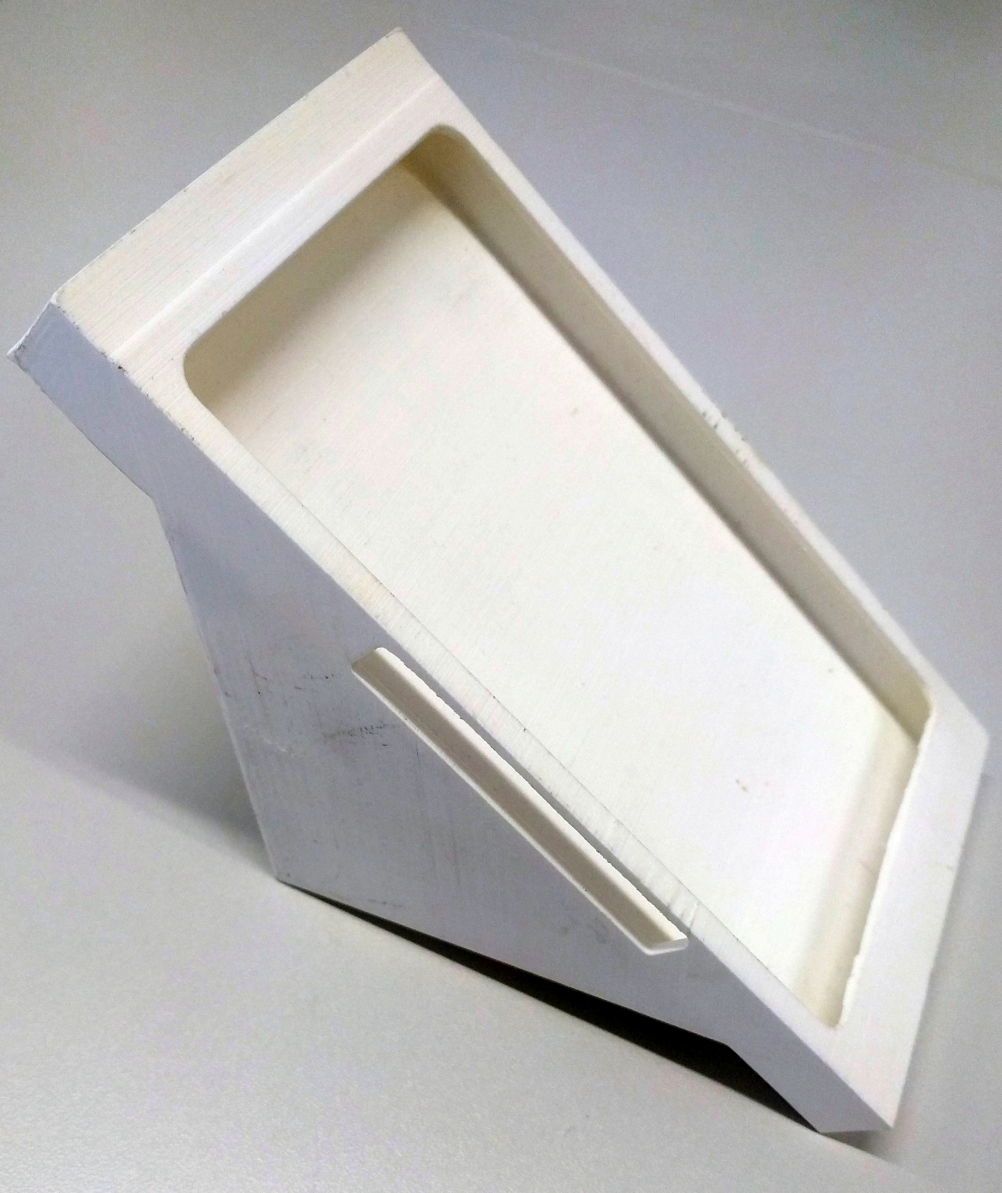
Measuring equipment. 3-D printed Smartphone Holder.

Two 3D-printed containers served as smartphone holders as shown in Fig 1. The holders were firmly attached to the weight stack using four strong neodym magnets (Webcraft AG, Uster, Switzerland). The magnets had a single adhesive force of 37.8 N. Per smartphone holder the four magnets exerted a force of totalling 151 N.

During the exercises, the magnet-equipped smartphone holders were attached to the weight stacks of the resistance exercise machines.

### Exercises

Before starting with the measurements, participants were shown all nine exercise machines. Correct settings and range of motion were determined according to participants individual anatomy. The participants were familiarized with the motor tasks to be performed on all of the resistance exercise machines. Next, the participants underwent a five minutes warm-up on a spinning bike (Schwinn, Vancouver, USA).

After the warm-up, the one repetition maximum (1-RM) was determined submaximally. Briefly, participants were asked to choose a resistance level they thought they could lift ten times maximally. Before starting the 1-RM assessment, participants were instructed to lift over the full range of motion. Only repetitions fulfilling this criterion were counted. If the chosen resistance that was lifted was more than four but less than ten times, 1-RM was extrapolated, using the formula described in Mayhew et al. [31]. If more than ten repetitions were achieved, the exercise was repeated with 20% increase of resistance, following a two minutes recovery break. This was repeated until the number of repetitions was in the defined range. After the 1-RM determination, participants performed two sets of ten repetitions with 60% of their 1-RM on all nine resistance exercise machines with a two minutes break in between sets and exercises. To ensure a real-world approach, the velocity of contractions were user-determined.

All exercises were recorded with a 62 mm lens Sony HDR-CX900E (Sony, Tokio, Japan) on a tripod using a resolution of 1920 x1080 pixels at 50 frames per second. Hence, the sampling frequency between smartphone accelerometer derived measurements and video recordings were different (400 Hz *vs* 50 Hz). However, we do not consider this discrepancy to be a limitation, because method-comparison studies with handheld devices *versus* machines, *e*.*g*. in dynamometry, will never be able to achieve synchronization nor sampling frequency equality [32].

### Rating

#### Video recordings

The free software Kinovea V0.8.27 (www.kinovea.org) was used for reviewing and rating the video recordings. Kinovea is a video player that is generally used for sports analysis. It allows frame-by-frame playback and includes a stopwatch function, which allows for precise annotation of specific time-critical events such as contraction-phases.

**Fig 2:**
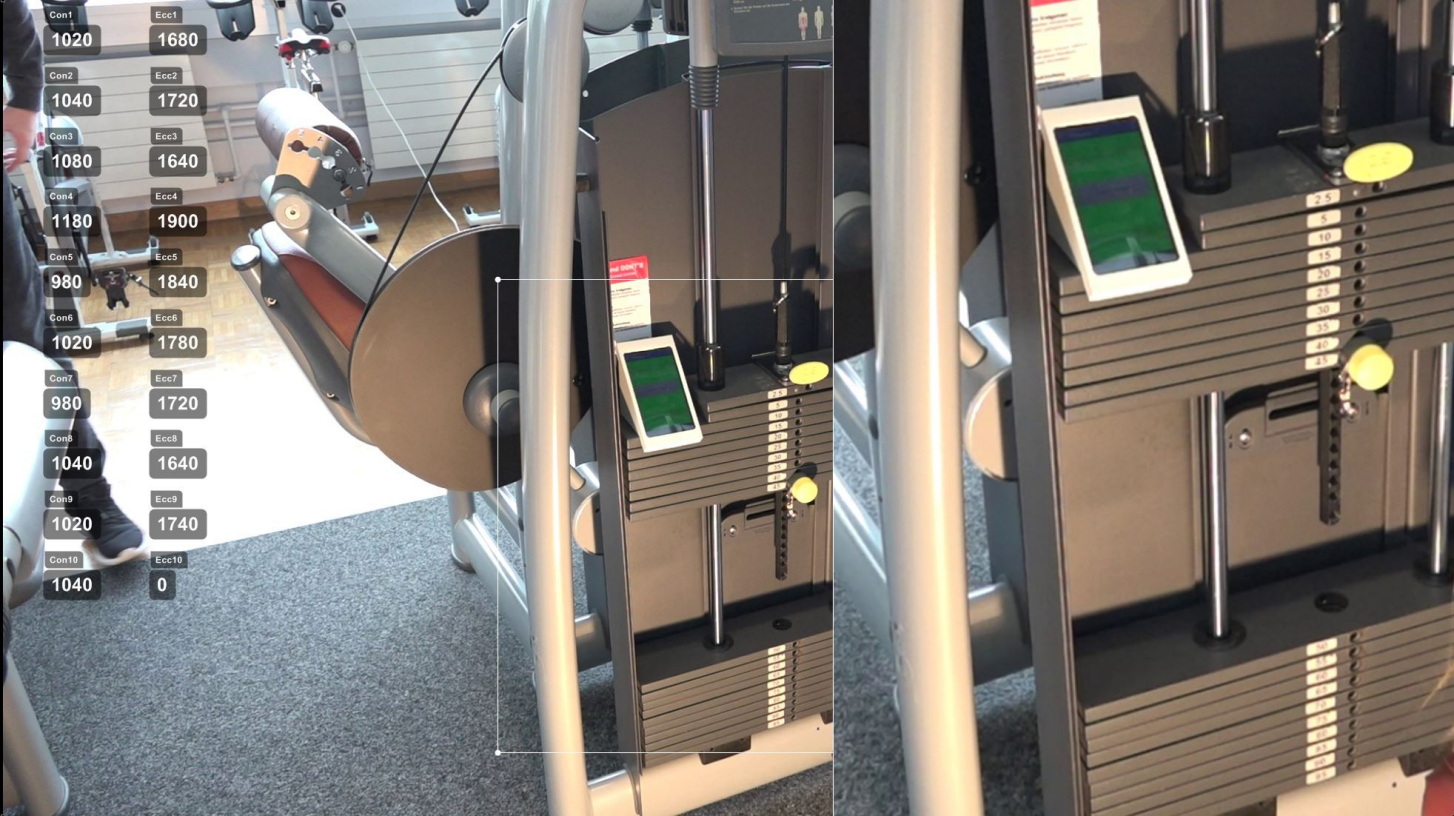
Rating of video recordings. Time per contraction phase was annotated in milliseconds using a 2.5x magnification of the weight stack.

Video recordings were rated by the two study investigators, who screened all recordings independently, frame-by-frame. A 2.5-fold magnification of the weight stack within Kinovea was used to determine contraction phases. The starting point of a concentric contraction was determined as the last frame before the weight stack movement was visually detected. The end of the concentric phase was defined as the first frame, whereby no additional increase of the weight stack could be visually recognized. This frame, due to the dynamic nature of the exercise, was then selected as the starting point of the eccentric phase. The endpoint of the eccentric phase was set to the last frame before the opposite weight stack movement was noticeable. All ten repetitions (20 contraction phases) were annotated in milliseconds in Kinovea as depicted in Fig 2.

#### Smartphone accelerometer derived data

Smartphone accelerometer derived data were analyzed using Matlab R2018a (The Mathworks, Nattick, USA). An algorithm was written with the specific aim to detect the number of repetitions, contraction-specific phases TUT and total TUT (see description of the algorithm in Supporting Information) generically. Briefly, the vector length was used, and data were pre-processed by applying a Hampel filter to remove outliers [33]. Non-unique timestamps were removed, and the data were subjected to interpolation to achieve an equidistant time series. The gravitational offset was subtracted. Repetition counting was performed by the single integration of the time series. The resulting drift was compensated by a polynomial fit. A moving average filter ensured curve smoothness. Thresholds for minimum inter-repetition distance and prominence were defined for peak detection on the integrated time series. Contraction-specific TUT was determined using the velocity curve zero-crossings.

The following numbers of variables were extracted from the video recordings and accelerometer data: (I) the number of single repetitions, (II) contraction-specific phases TUT, (III) temporal length of single repetitions as the sum of the concentric and eccentric phase TUT and (IV) the total TUT, which is defined as the sum of all repetitions TUT (10) during a set [26].

### Data and statistical analyses

#### Validity

The analysis aimed to determine whether crucial mechano-biological resistance exercise descriptors including the number of single repetitions, contraction-specific phases TUT and the total TUT can be identified reliably from smartphone accelerometer data. Both raters examined video recordings independently and in a randomized order. For the method comparison, the mean of the video recording results and the mean of the algorithmic detection of the two smartphones derived accelerometer data were calculated.

Bland-Altman plots were used to compare the two methods visually. Systematic bias is depicted by the mean difference between the two methods. To examine the linear association between the methods, Pearson correlation coefficients were calculated. Limits of agreement (LoA) were used to determine the level of agreement between methods [34]. The LoA for all contraction phases was calculated as the mean difference between methods, whereby 2.5% or 97.5% denoted the lower and upper limits, respectively [35]. The normalized error was calculated as the division of the contraction-specific mean of the differences between the two methods and the contraction-specific TUT of the algorithmic rating [34].

Methodological outlier removal was performed as described for exploratory studies in [36]. To summarize, the interquartile range (IQR) of the mean difference of the two methods was calculated per contraction-specific phase for every resistance exercise machine. Data greater than 1.5 or smaller than -1.5 times the IQR were marked and excluded, as suggested by Sachs and Hedderich [36]. Visual assessment of heteroscedasticity was performed without recognizing trends towards heteroscedasticity.

#### Scoring reliability of the two raters

Interrater reliability and agreement were examined between the two raters who rated all 18 sets, consisting of ten repetitions each, on nine resistance exercises machines of 22 participants. The raters annotated all TUT of all contraction-specific phases. Interrater reliability was calculated using a two-way random-effects model (2.1), single measures, absolute agreement and ICC.

## Results

The algorithmic detection of single repetitions derived from the two smartphones accelerometers yielded high precision, recall and accuracy. Mean precision was 0.9972 ± 0.0000 (mean ± SD) for both smartphones. The average accuracy, calculated by the F-Score, for all the exercise machines, was 0.9948 ± 0.0004 (mean ± SD), which equals an error rate of 0.16%.

**Fig 3:**
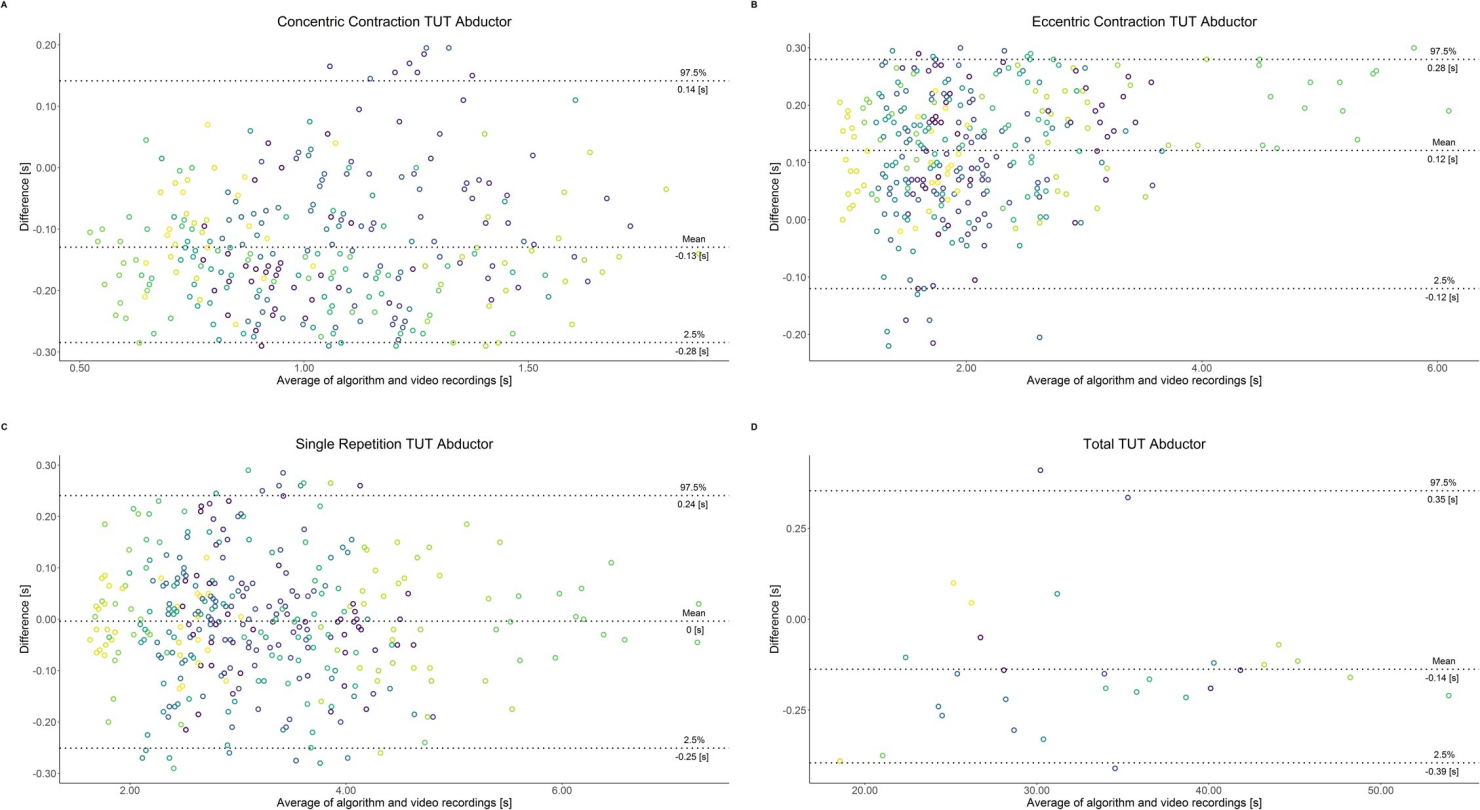
Bland-Altman plots of agreement between algorithmically accelerometer derived and video recordings derived contraction-specific phases of the Abductor machine. A: Concentric contraction phase. B: Eccentric contraction phase. C: Single repetition. D: Total time-under-tension.

**Fig 4:**
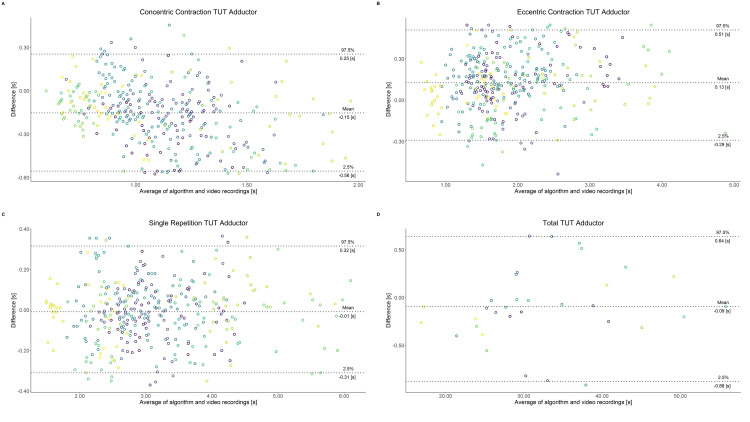
Bland-Altman plots of agreement between algorithmically accelerometer derived and video recordings derived contraction-specific phases of the Adductor machine. A: Concentric contraction phase. B: Eccentric contraction phase. C: Single repetition. D: Total time-under-tension.

**Fig 5:**
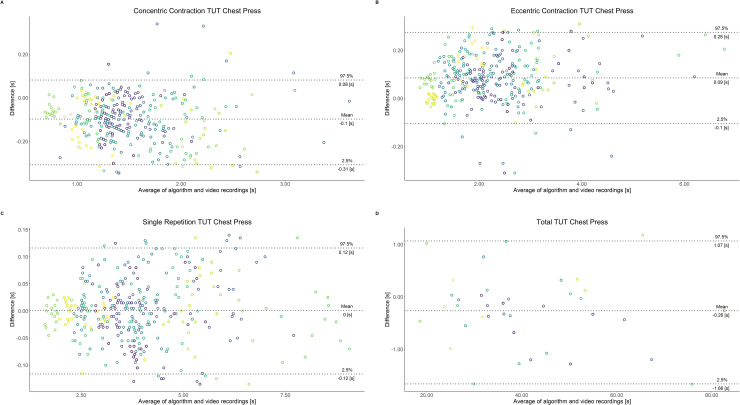
Bland-Altman plots of agreement between algorithmically accelerometer derived and video recordings derived contraction-specific phases of the Chest Press machine. A: Concentric contraction phase. B: Eccentric contraction phase. C: Single repetition. D: Total time-under-tension.

**Fig 6:**
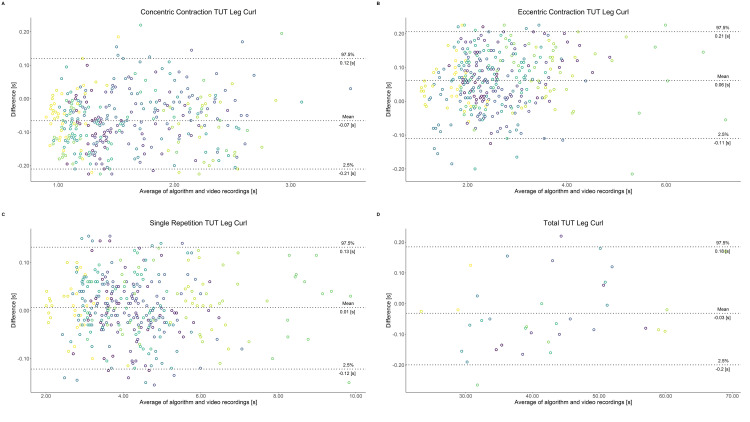
Bland-Altman plots of agreement between algorithmically accelerometer derived and video recordings derived contraction-specific phases of the Leg Curl machine. A: Concentric contraction phase. B: Eccentric contraction phase. C: Single repetition. D: Total time-under-tension.

**Fig 7:**
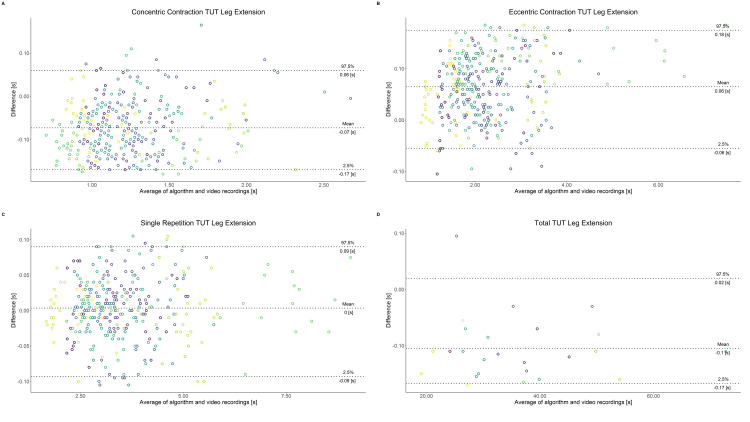
Bland-Altman plots of agreement between algorithmically accelerometer derived and video recordings derived contraction-specific phases of the Leg Extension machine. A: Concentric contraction phase. B: Eccentric contraction phase. C: Single repetition. D: Total time-under-tension.

**Fig 8:**
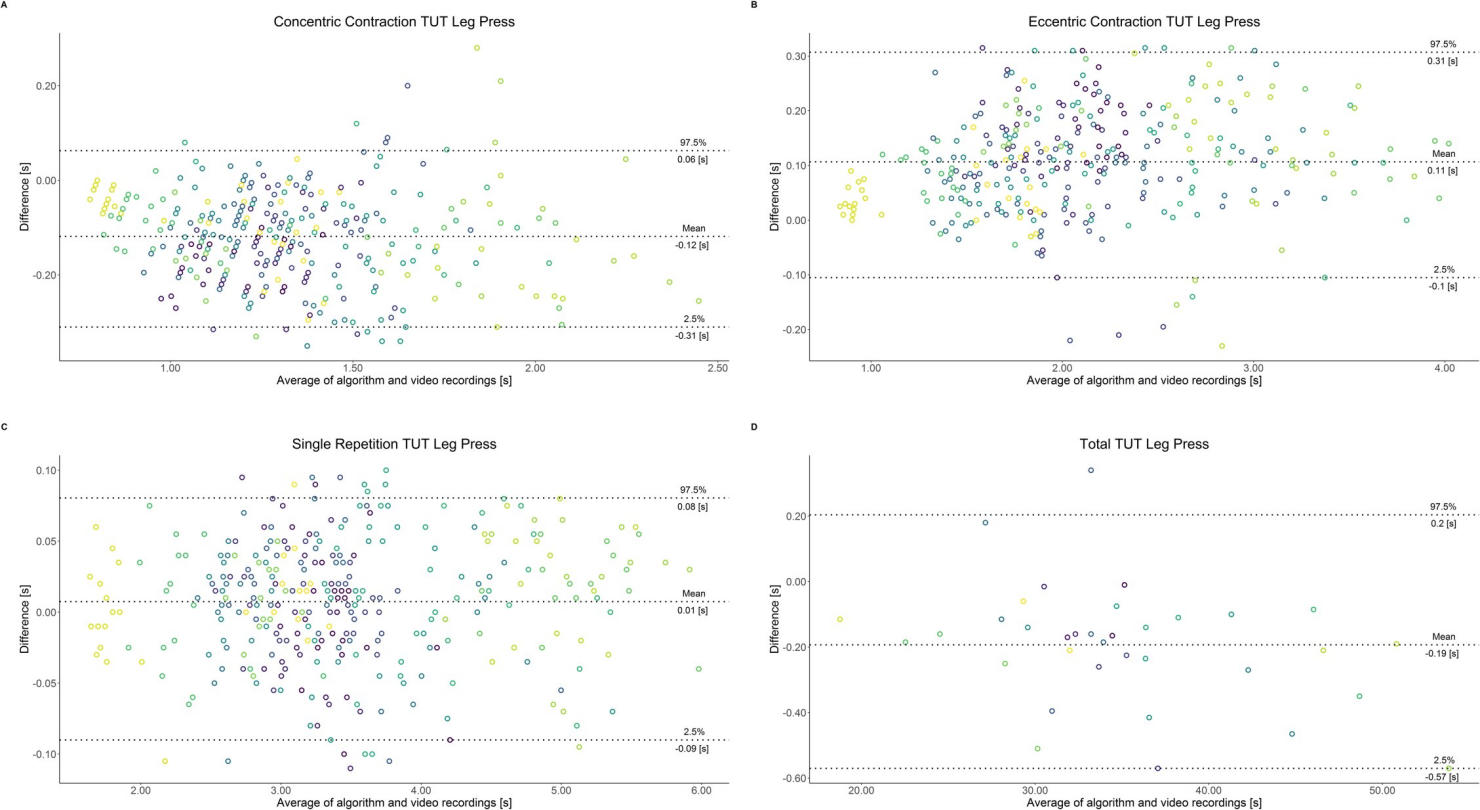
Bland-Altman plots of agreement between algorithmically accelerometer derived and video recordings derived contraction-specific phases of the Leg Press machine. A: Concentric contraction phase. B: Eccentric contraction phase. C: Single repetition. D: Total time-under-tension.

**Fig 9:**
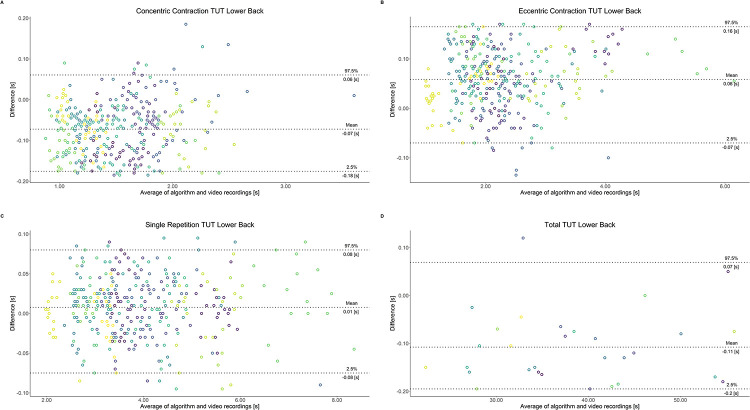
Bland-Altman plots of agreement between algorithmically accelerometer derived and video recordings derived contraction-specific phases of the Lower Back machine. A: Concentric contraction phase. B: Eccentric contraction phase. C: Single repetition. D: Total time-under-tension.

**Fig 10:**
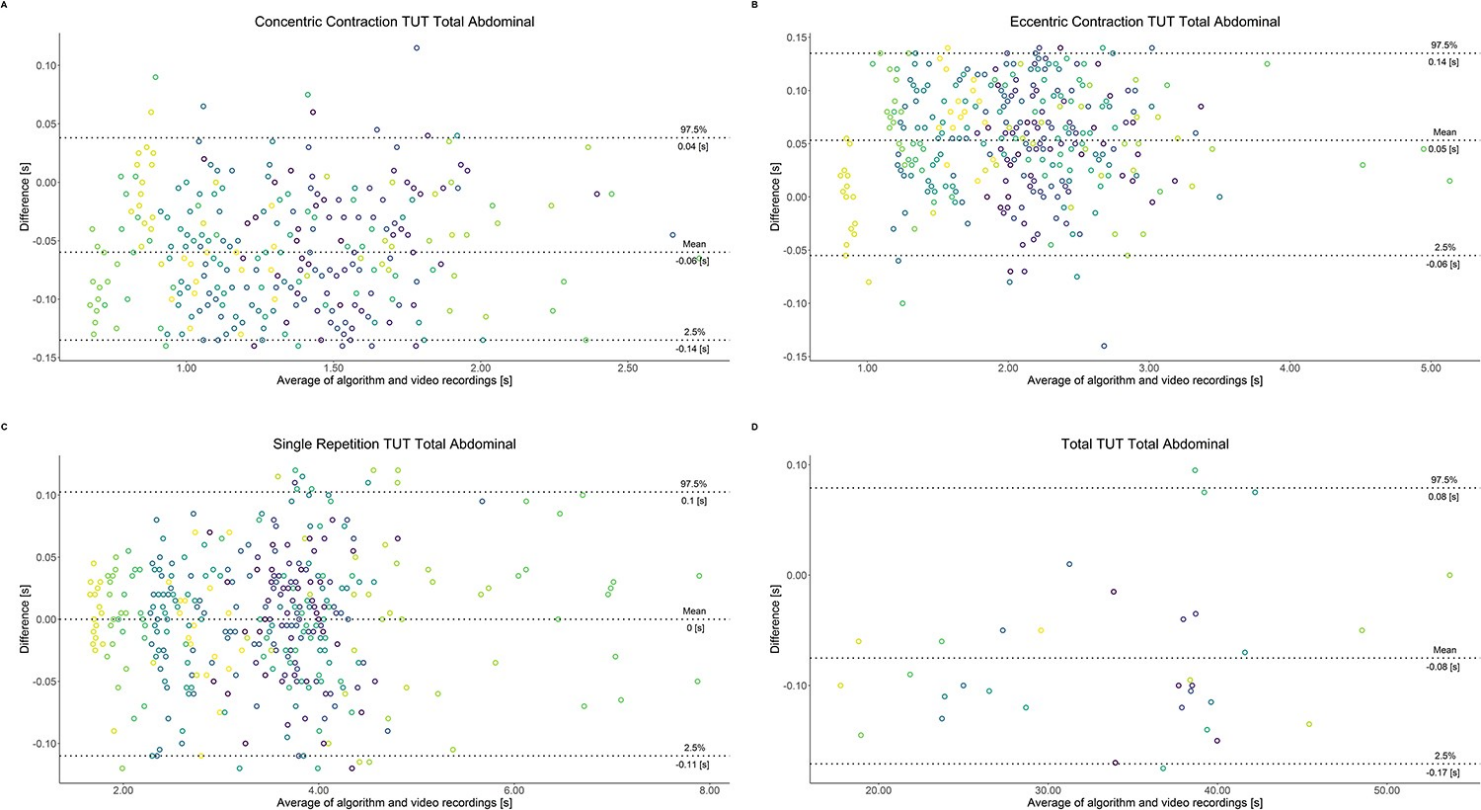
Bland-Altman plots of agreement between algorithmically accelerometer derived and video recordings derived contraction-specific phases of the Total Abdominal machine. A: Concentric contraction phase. B: Eccentric contraction phase. C: Single repetition. D: Total time-under-tension.

**Fig 11:**
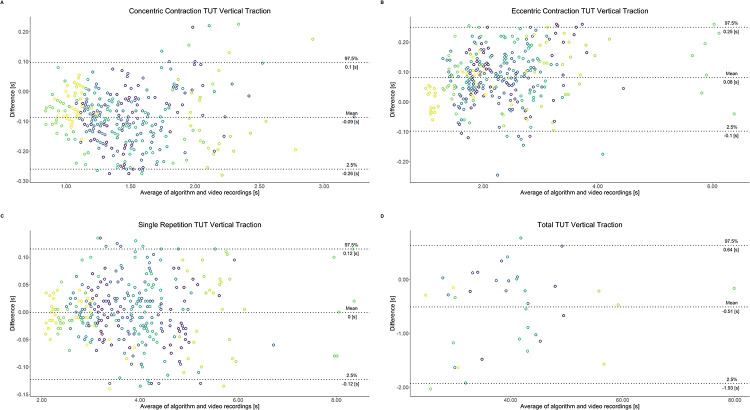
Bland-Altman plots of agreement between algorithmically accelerometer derived and video recordings derived contraction-specific phases of the Vertical Traction machine. A: Concentric contraction phase. B: Eccentric contraction phase. C: Single repetition. D: Total time-under-tension.

**Fig 12:**
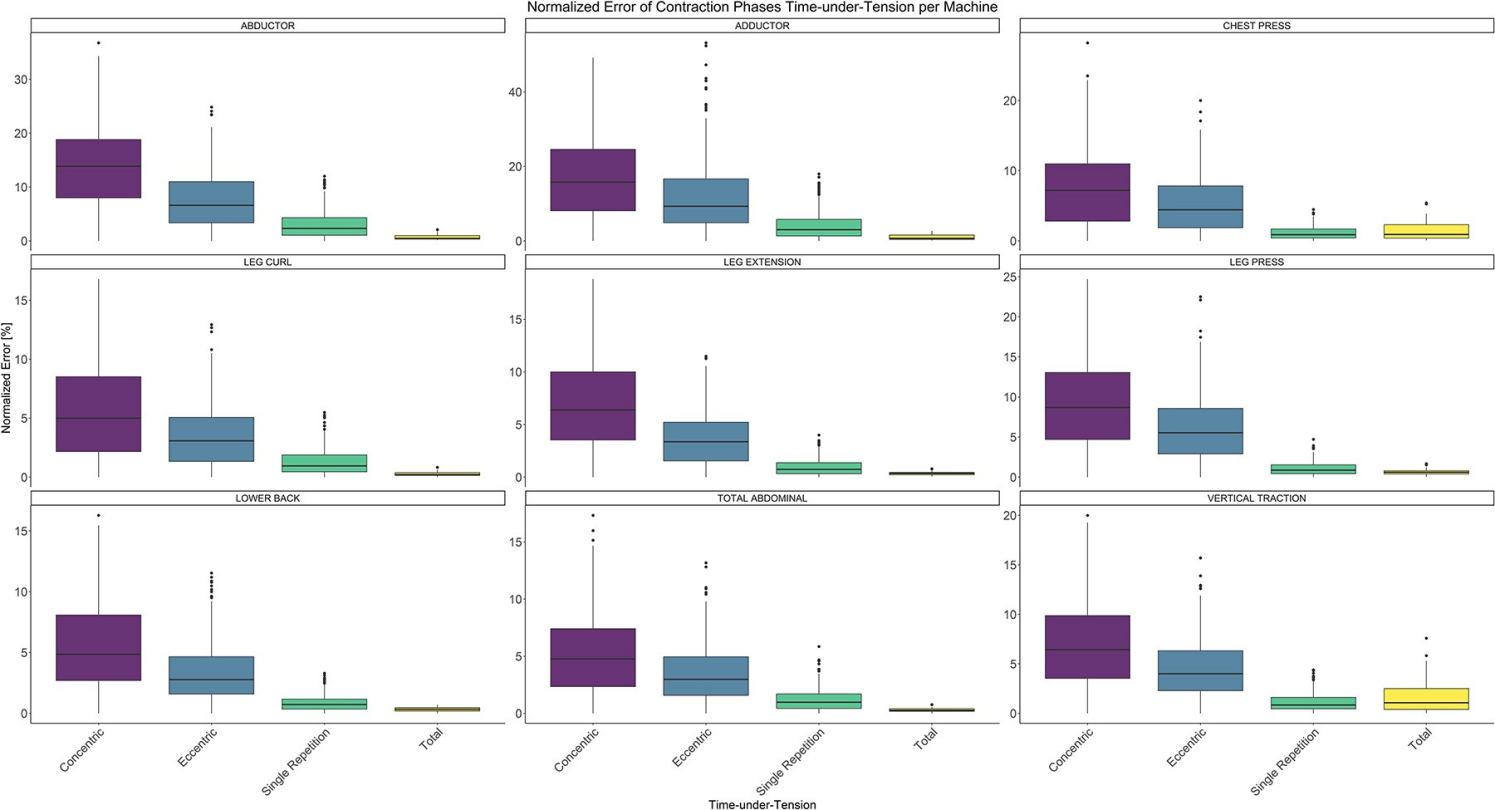
Boxplot of normalized errors of contraction-specific phases for all resistance exercise machines.

Comparing video recordings to algorithmically-derived, contraction-phase specific TUT, showed a high degree of correlation (r > 0.93) for all exercise machines (Table 1). Additionally, the ICC for the interrater reliability was above 0.99 with 95% CI [0.99, 1.00] for all contraction-phase specific TUT. Notably, concentric contraction-specific TUT (median = -0.09 s) was hereby systematically overestimated while eccentric contraction-specific TUT (median = 0.08 s) was systematically underestimated by the algorithm (Z = -55.49, *p* < 2.2−^16^). Table 2 shows agreement between concentric, eccentric, single repetition and total time-under-tension derived from algorithmic accelerometer data and video recordings. In Figs 3–11 Bland-Altman plots visualize the systematic bias as the mean difference between the methods whereas Fig 12 depicts the normalized errors of contraction-specific phases for all resistance exercise machines.

**Table 1:** Agreement between concentric, eccentric, single repetition and total time-under-tension, derived from algorithmic accelerometer data and video recordings.

Machine	Mean time based on accelerometer data [s] (±SD)	Mean time based on video recordings [s] (±SD)	Mean difference [s] (±SD)	Correlation	95% Limits of Agreement [s]	Agreement (% of mean TUT from algorithm)
**Abductor**						
Concentric TUT (*n* = 3114)	1.10 (0.28)	0.98 (0.29)	-0.13 (0.10)	0.93*	-0.28–0.14	11.71
Eccentric TUT (*n* = 3114)	2.04 (0.87)	2.17 (0.89)	0.12 (0.11)	0.99*	-0.12–0.28	5.92
Single Repetition TUT (*n* = 3180)	3.23 (1.03)	3.23 (1.02)	0.00 (0.12)	0.99*	-0.25–0.24	0.11
Total TUT (*n* = 298)	33.51 (8.63)	33.37 (8.64)	-0.14 (0.18)	1.00*	-0.39–0.35	0.41
**Adductor**						
Concentric TUT (*n* = 3114)	1.18 (0.33)	1.03 (0.26)	-0.15 (0.21)	0.78*	-0.56–0.25	12.93
Eccentric TUT (*n* = 3137)	1.83 (0.67)	1.96 (0.70)	0.13 (0.20)	0.96*	-0.29–0.51	6.94
Single Repetition TUT (*n* = 3180)	3.12 (0.92)	3.11 (0.92)	-0.01 (0.15)	0.99*	-0.31–0.32	0.26
Total TUT (n = 298)	32.55 (9.26)	32.46 (9.33)	-0.09 (0.39)	1.00*	-0.88–0.64	0.29
**Chest Press**						
Concentric TUT (*n =* 3114)	1.54 (0.47)	1.44 (0.46)	-0.1 (0.11)	0.97*	-0.31–0.08	6.32
Eccentric TUT (*n =* 3137)	2.21 (0.93)	2.29 (0.95)	0.09 (0.10)	0.99*	-0.10–0.28	3.90
Single Repetition TUT (*n =* 3180)	3.92 (1.45)	3.93 (1.45)	0.00 (0.06)	1.00*	-0.12–0.12	0.03
Total TUT (*n =* 298)	40.94 (15.45)	40.67 (15.34)	-0.27 (0.71)	1.00*	-1.66–1.07	0.67
**Leg Curl**						
Concentric TUT (*n =* 3114)	1.63 (0.50)	1.57 (0.51)	-0.07 (0.08)	0.99*	-0.21–0.12	4.03
Eccentric TUT (*n =* 3137)	2.57 (0.90)	2.64 (0.92)	0.06 (0.08)	1.00*	-0.11–0.21	2.39
Single Repetition TUT (*n =* 3180)	4.32 (1.40)	4.33 (1.40)	0.01 (0.06)	1.00*	-0.12–0.13	0.15
Total TUT (*n =* 298)	42.29 (10.66)	42.25 (10.70)	-0.03 (0.12)	1.00*	-0.20–0.18	0.07
**Leg Extension**						
Concentric TUT (*n =* 3114)	1.27 (0.31)	1.19 (0.32)	-0.07 (0.06)	0.98*	-0.17–0.06	5.78
Eccentric TUT (*n =* 3137)	2.21 (0.92)	2.27 (0.94)	0.07 (0.06)	1.00*	-0.06–0.18	2.96
Single Repetition TUT (*n =* 3180)	3.57 (1.24)	3.57 (1.24)	0.00 (0.04)	1.00*	-0.09–0.09	0.09
Total TUT (*n =* 298)	35.89 (12.31)	35.79 (12.31)	-0.10 (0.06)	1.00*	-0.17–0.02	0.29
**Leg Press**						
Concentric TUT (*n =* 3114)	1.39 (0.32)	1.27 (0.31)	-0.12 (0.10)	0.95*	-0.31–0.06	8.59
Eccentric TUT (*n =* 3137)	2.05 (0.66)	2.15 (0.67)	0.11 (0.10)	0.99*	-0.10–0.31	5.25
Single Repetition TUT (*n =* 3180)	3.43 (0.90)	3.44 (0.91)	0.01 (0.04)	1.00*	-0.09–0.08	0.22
Total TUT (*n =* 298)	35.2 (7.89)	35.00 (7.82)	-0.19 (0.18)	1.00*	-0.57–0.2	0.55
**Lower Back**						
Concentric TUT (*n =* 3114)	1.54 (0.38)	1.47 (0.39)	-0.07 (0.06)	0.99*	-0.18–0.06	4.68
Eccentric TUT (*n =* 3137)	2.25 (0.80)	2.31 (0.81)	0.06 (0.06)	1.00*	-0.07–0.16	2.60
Single Repetition TUT (*n =* 3180)	3.93 (1.21)	3.93 (1.21)	0.01 (0.04)	1.00*	-0.08–0.08	0.20
Total TUT (*n =* 298)	38.39 (9.32)	38.28 (9.33)	-0.11 (0.07)	1.00*	-0.20–0.07	0.28
**Total Abdominal**						
Concentric TUT (*n =* 3114)	1.37 (0.37)	1.31 (0.38)	-0.06 (0.05)	0.99*	-0.14–0.04	4.38
Eccentric TUT (*n =* 3137)	2.01 (0.65)	2.06 (0.65)	0.05 (0.05)	1.00*	-0.06–0.14	2.65
Single Repetition TUT (*n =* 3180)	3.46 (1.10)	3.46 (1.10)	0.00 (0.05)	1.00*	-0.11–0.10	0.00
Total TUT (*n =* 298)	33.9 (8.92)	33.83 (8.94)	-0.08 (0.07)	1.00*	-0.17–0.08	0.22
**Vertical Traction**						
Concentric TUT (*n =* 3114)	1.50 (0.38)	1.41 (0.39)	-0.09 (0.09)	0.97*	-0.26–0.10	5.78
Eccentric TUT (*n =* 3137)	2.26 (0.81)	2.34 (0.82)	0.08 (0.09)	0.99*	-0.10–0.23	3.52
Single Repetition TUT (*n =* 3180)	3.82 (1.10)	3.82 (1.10)	0.00 (0.06)	1.00*	-0.12–0.12	0.02
Total TUT (*n =* 298)	40.45 (10.82)	39.9 (10.90)	-0.55 (0.73)	1.00*	-1.93–0.46	1.35

Abbreviations: TUT: time-under-tension.

* Denotes: *p* < 0.00001.

**Table 2:** Anthropometrical information of participants.

	Young (*n* = 18)	Old (*n* = 4)	Total (*n* = 22)	*p* value
**Age [years]**				< 0.001
Mean (SD)	36.2 (9.1)	65.5 (4.8)	41.5 (14.3)	
Range	19–51	6–70	19–70	
**Sex**				0.746
Male	12 (66.7%)	3 (75.0%)	15 (68.2%)	
Female	6 (33.3%)	1 (25.0%)	7 (31.8%)	
**Weight [kg]**				0.891
Mean (SD)	77.78 (16.9)	76.5 (14.8)	77.5 (16.2)	
Range	46–105	57–91	46–105	
**Height [m]**				0.982
Mean (SD)	1.75 (0.1)	1.75 (0.1)	1.754 (0.1)	
Range	1.60–1.87	1.70–1.81	1.60–1.87	

Young refers to the population younger than 60 while old includes 60 years and older.

Additionally, single contraction-specific phases were compared using a Mann Withney U test between young and old participants on all the exercise machines (Table 3).

**Table 3:** Comparison of contraction-specific phases between young and old participants.

		Age			
		Young (< 60 years, *n* = 18)	Old (> = 60 years, *n* = 4)		
Machine	Phase	Median [s]	Median [s]	U	*p* value
Abductor	Con	0.94	0.99	12317	0.043*
Abductor	Ecc	2.00	1.91	15850	0.159
Abductor	Rep	3.06	2.86	14726	0.752
Abductor	TuT	30.21	29.37	146.5	0.951
Adductor	Con	0.97	1.05	11553	0.006**
Adductor	Ecc	1.95	1.90	16999.5	0.012*
Adductor	Rep	2.96	2.95	15787	0.178
Adductor	TuT	29.72	30.17	157	0.704
Chest Press	Con	1.29	1.71	7111	0.000***
Chest Press	Ecc	2.43	2.62	13497.5	0.381
Chest Press	Rep	3.74	4.33	11118.5	0.001**
Chest Press	TuT	37.24	43.43	108	0.280
Leg Curl	Con	1.33	1.93	6905.5	0.000***
Leg Curl	Ecc	2.45	2.66	14134.5	0.894
Leg Curl	Rep	4.05	4.79	11073.5	0.004**
Leg Curl	TuT	39.88	50.63	104	0.268
Leg Extension	Con	1.09	1.31	7700.5	0.000***
Leg Extension	Ecc	2.13	2.51	12664.5	0.092
Leg Extension	Rep	3.31	3.93	11072.5	0.001**
Leg Extension	TuT	33.58	41.19	103	0.218
Leg Press	Con	1.20	1.27	12158.5	0.029*
Leg Press	Ecc	2.16	2.21	14800.5	0.697
Leg Press	Rep	3.39	3.66	14042.5	0.729
Leg Press	TuT	33.79	34.88	139	0.891
Lower Back	Con	1.43	1.56	11985	0.019*
Lower Back	Ecc	2.23	2.42	13613.5	0.445
Lower Back	Rep	3.66	4.19	12587.5	0.078
Lower Back	TuT	36.90	43.66	125	0.573
Total Abdominal	Con	1.33	1.32	12697	0.098
Total Abdominal	Ecc	2.15	2.35	12718.5	0.102
Total Abdominal	Rep	3.61	3.78	12539.5	0.071
Total Abdominal	TuT	37.18	39.01	114.5	0.378
Vertical Traction	Con	1.34	1.52	10571.5	0.000***
Vertical Traction	Ecc	2.48	2.23	15703	0.205
Vertical Traction	Rep	3.92	3.83	14257	0.890
Vertical Traction	TuT	39.37	41.80	134	0.773

*n* = 22 participants; Con: concentric phase TuT; Ecc: eccentric phase TuT; Rep: single repetition TuT; TuT: total time-under-tension.

* Denotes: *p* < 0.05.

** Denotes: *p* < 0.01.

*** Denotes: *p* < 0.001.

## Discussion

In this study, we show that mechano-biological descriptors such as single repetitions, the temporal distribution of contraction-specific phases and total time-under-tension can be reliably and validly extracted from smartphone accelerometer-derived data.

Evidence for this finding is that the error for single repetition detection is 0.16% when compared to the associated video recordings that represented the gold standard. A multi-analytical, algorithmic approach achieves the reported error for single repetition detection of 0.16%. The mean temporal error of single repetitions, when compared to the gold standard, is 0.12%. Theoretically, three different domains could be used for detecting single repetitions from the accelerometer data. These domains are the acceleration, the velocity and the displacement domain. We noticed, that the signal-to-noise ratio, even when algorithmically preprocessing the accelerometer data, was not sufficient to generically detect single repetitions over a wide range of user-exerted accelerations. Using displacement as the single repetition extracting domain was not an option, because double integration amplifies any offsets, non-linearities, and noise. Therefore, the velocity domain was chosen to extract single repetitions. Accordingly, after preprocessing, accelerometer data was integrated (Eq 1). Moreover, the mean differences for concentric contractions TUT on nine different resistance exercise machines ranged from -0.15 to—0.07 s. Rathleff et al. [37] found the mean difference between stretch-sensor data and video recordings for concentric contractions TUT to be 0.09 s with 95% CI [0.06 s, 0.11 s]. Hence, the findings of Rathleff et al. [37] corresponded with our results.

Due to the fact that participants were allowed to choose their individual, contraction-specific resistance exercise velocity, concentric contractions TUT were significantly lower (1.30 ± 0.40 s, mean ± SD) than eccentric contractions TUT (2.24 ± 0.84 s, mean ± SD) in our study (Z = -50739, *p* < 2.2^−16^). The systematic lower concentric contractions TUT increased the normalized error, while increasing TUT (*e*.*g*. as seen for eccentric contractions TUT, single repetitions TUT or total TUT) decreased the normalized error, as also reported in Rathleff et al. [37].

v=∫adt (1)

We detected a systematic bias where concentric contractions TUT were overestimated by the algorithmic detection, while eccentric contractions TUT were slightly underestimated. This can be explained, in part, by the interpolation and drift-compensating polynomial fit used in the algorithm and/or the rating method of the video recordings. Additionally, time-mapping contraction-specific phases, as suggested in the methods, led to an overestimation of eccentric contractions TUT of the video recordings. Therefore, it is plausible that the systematic underestimation of the eccentric contraction TUT by the algorithm is caused by the slight overestimation of the eccentric contractions TUT of the video recordings rating. We are well aware of the fact that participants could potentially briefly rest at reversal points of contractions resulting in short isometric phases. Isometric contractions, no shortening or lengthening of muscle fibers, result in zero momentum, thus posing analytical difficulties when analyzing such phases algorithmically. Rathleff et al. [37], in Fig 4, described a quasi-isometric phase after a concentric contraction which shows a negative slope and could therefore, by a strict definition of muscle actions [38], be assigned to the eccentric contraction phase. Thus, we decided that a concentric contraction phase is followed by an immediate eccentric contraction phase, as described in the methods. However, we are convinced that this slight algorithmic underestimation of the eccentric phase TUT is not of clinical relevance.

We know that using the weight stack of resistance exercise machines as a surrogate for skeletal muscle contraction-specific phases does not necessarily coincide with contraction-specific phases of the targeted muscle fibers. A previous study dealing with high frame rate ultrasound, revealed that the onset of actual sarcomeric contraction of muscle fibers starts before the onset of force generation [39]. In young and healthy controls (*n* = 13, age range: 6–24 years), the force transmission time delay was measured with 0.008 ± 0.002 s [39]. Additionally, our resistance exercise machines, traditionally used cable pulls for force transmission. Hence, material properties of cable pulls (*e*.*g*. sloppiness of mounting, amount of play, *etc*.) could introduce additional temporal delays. Although temporal mapping of resistance exercise weight stack movement does not precisely reflect skeletal muscle contraction-specific phases, we have determined that these small temporal differences are not of clinical relevance.

Finally, TUT reflects the summation of single repetitions. Subsequently, the normalized error of the total TUT was 0.46%. Pernek et al. [40] found a temporal error of exercise duration of about 11%. Because different algorithmic approaches were used, dynamic-time-warping (calibration repetitions mandatory) [41] *vs*. a multi-analytical approach, a direct methodological comparison is difficult. Nonetheless, a multi-analytical algorithmic approach yielded a higher level of accuracy for measuring the total TUT on machine-based resistance exercises. Moreover, the time effort for end users is minimized because no calibration repetitions are necessary.

Comparing contraction-phase specific TUT between young and old participants revealed that young participants seemed to have a systematic, statistically significant lower median contraction-phase specific TUT when looking at the contraction phases that revealed a significant difference. An exception was found when looking at the adductor machine where old participants had a lower TUT during the eccentric phase. Hence, one-quarter of the measurements showed significant differences of contraction-specific TUT whereas the data show a tendency towards lower TUT for the young participants. To investigate this interesting aspect, a future study design should include the harvesting of biopsies to examine fiber type distribution. It has been shown that with increasing age the rate of force development declines [42]. Therefore, our results hint in this direction.

### Practical relevance of the results

Systematic reporting of all resistance exercise mechano-biological descriptors, as postulated by Toigo et al. [26], makes musculo-skeletal adaptions comparable. It did not escape our notice that our algorithmic approach could help to standardize resistance exercise reporting. We showed that off-the-shelf smartphones could be used to extract contraction-specific mechano-biological descriptors from user-exerted accelerations on a weight stack, during the time a participant worked out on a resistance exercise machine. The approach of using the acceleration vector length allows the recording smartphone(s) to be placed in any arbitrary orientation on the weight stack. This simple method not only enables researchers to standardize resistance exercise reporting, but also enables clinicians, sports professionals, and/or end-users to record, evaluate and/or compare resistance exercise mechano-biological descriptors.

Using this simple tool, healthcare professionals could monitor patient’s resistance exercise training as well as decreasing patient-self reporting burden. As such, rehabilitation protocols could then be individually adjusted.

Due to the general ageing trends of the population, standardized reporting has been found to be important for personal resistance exercise interventions combating and/or reversing sarcopenia.

### Limitations

As Pernek et al. [40] tested smartphone accelerometer-derived weight stack data with different weights, 50% 1-RM for the first set and 70% 1-RM for the second set of ten repetitions, we focused on deeper data analytical insights *i*.*e*. the extraction of mechano-biological descriptors.

The study was designed to reflect a real-world resistance exercise training where the participants determined contraction velocities. Although this study design permitted the collection of considerable intra- and interindividual variation of acceleration and/or velocity resistance exercise data, it does not permit testing of the boundaries of the algorithm. Hence, as we focused on the extraction of mechano-biological descriptors, we could not test for any critical algorithmic boundaries.

Two Nexus 6P smartphones with built-in 3-axis accelerometer BMI160 (Robert Bosch GmbH, Stuttgart, Germany) were used in this study. Note that the operating system, Android (Open Handset Alliance, Maintain View, USA), is a non-real time operating system. Therefore, accelerometer-measured data values can be delayed, resulting in incorrect timestamps, or, in other instances, dropped, because the device is busy [43]. Dropping or making timestamps equidistant might also have contributed to the introduction of small random temporal errors.

Because smartphone accelerometers measure proper accelerations, contraction-specific phases of dynamic resistance exercises can validly and reliably be extracted from accelerometer data. However, temporal segments without proper acceleration cannot unequivocally be assigned to any contraction-specific phases, because they could belong to isometric contractions or dynamic, constant-velocity contractions. Therefore, in a real-world scenario, our algorithmic approach could be used, whereas for isometric contractions or constant velocity movements, caution is required.

As a gold standard, video recordings with a sampling frequency of 50 Hz was chosen. This is lower than the sampling frequency returned by the smartphone accelerometers, which was approximately 400 Hz. This fact may have led to the introduction of a random error, as reversal points between contraction phases might have been masked in between two frames of the video recording. Using displacement sensors might increase the precision of contraction phase mapping. However, given the high degree of agreement between the methods found in the current study, we are convinced that it will not be of clinical relevance.

### Future research

A future study should address the examination of algorithmic boundaries of resistance exercise mechano-biological descriptors extraction. Different contraction velocities at different loads (*e*.*g*. 30% *vs*. 90% 1-RM) should be tested to investigate the influence on the algorithm.

Using displacement sensors to detect weight stack movements has the potential to diminish systematic temporal bias of contraction-specific TUT, while allowing for the detection of isometric or quasi-isometric segments. As we examined accelerometer data derived from unidimensional weight stack movements, we determined that non-constraint environments, such as free weights, should also be investigated.

Citizen-science big data approaches have the potential to solve scientific questions. Here we showed that smartphones can be vectors for reliably and validly collecting and reporting machine-based resistance exercise data. Identifying and reporting postulated mechano-biological descriptors and/or methods both contribute to solving the dilemma of underreporting resistance exercise determinants. Therefore, distinct morphological, molecular and metabolic adaptations on the muscular level can be elucidated by off-the-shelf smartphone-based big data approaches.

## Supporting information

S1 Fig Representative smartphone recording of ten repetitions of a weight stack of a resistance exercise machine. A: Raw data acceleration profile, B: Velocity domain of single-integrated raw data profile. Triangles annotate peaks and red circles denote zero-crossings.
(DOCX)

S2 Fig Flow-chart of the algorithm. **Notation:** a(t) = acceleration; v(t) = velocity; fe = equidistant frequency; R = number of repetitions; ppp = position of positive peaks; zcp = zero crossing positions; pl = phase lengths.
(TIF)

S1 Code snippet Pseudo-code for single repetition detection and contraction-specific phase extraction. (DOCX)
