# Supplementary material for: Using smartphone accelerometer data to obtain scientific mechanical-biological descriptors of resistance exercise training
Source: PLoS One. 2020 Jul 15;15(7):e0235156. doi: 10.1371/journal.pone.0235156 (PMC7363108; doi:10.1371/journal.pone.0235156)
Supplement: S1 Fig — A: Raw data acceleration profile, B: Velocity domain of single-integrated raw data profile. Triangles annotate peaks and red circles denote zero-crossings. (DOCX) [file pone.0235156.s001.docx]

## Description of the algorithm


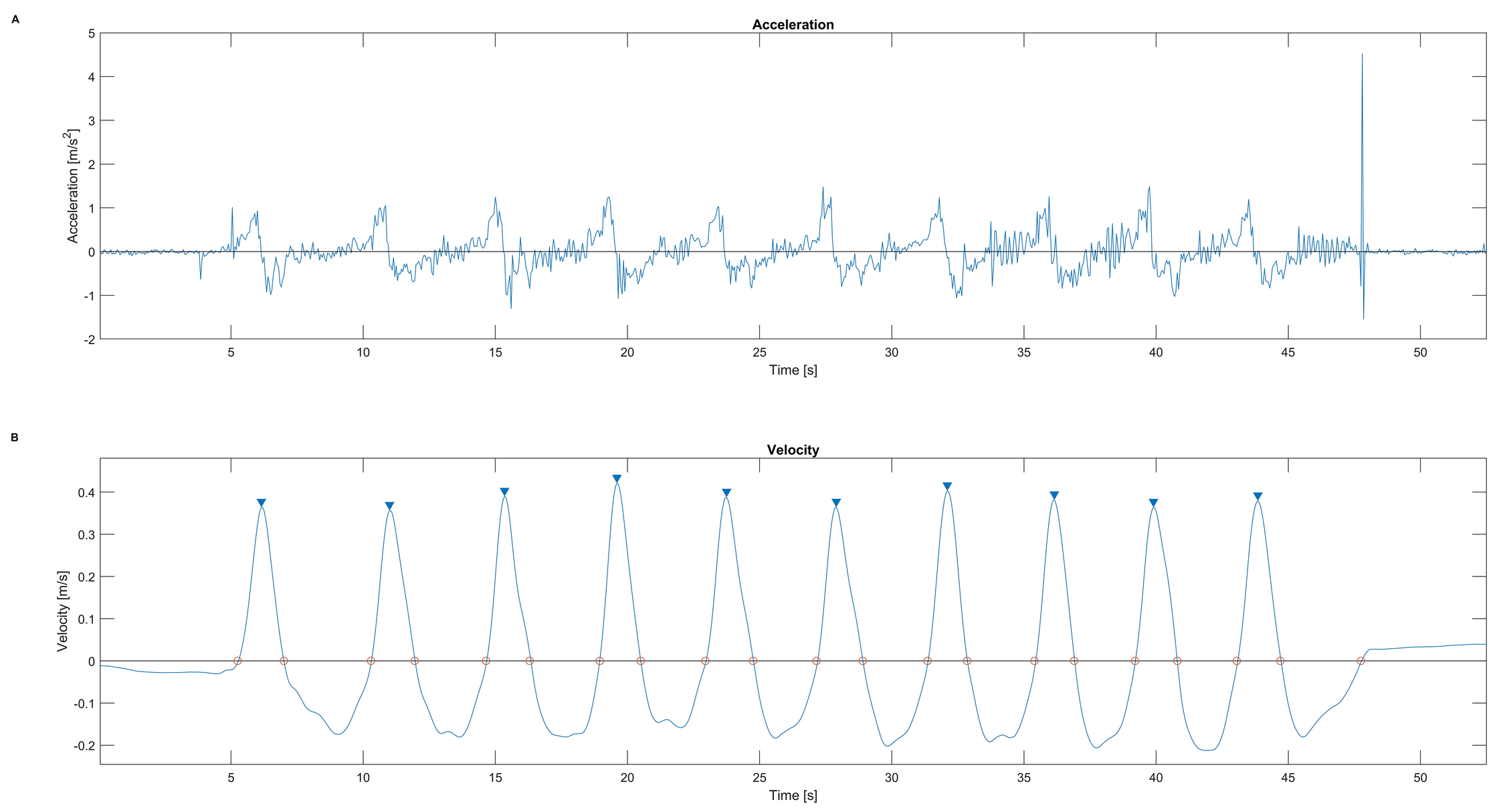


**S1 Fig. Representative smartphone recording of ten repetitions of a weight stack of a resistance exercise machine.** A: Raw data acceleration profile, B: Velocity domain of single-integrated raw data profile. Triangles annotate peaks and red circles denote zero-crossings.

The figures above are representative figures of our data collection. S1 Fig. A shows the acceleration raw data profile of ten repetitions on a resistance exercise machine collected by a single Nexus 6P smartphone. S1 Fig. B depicts the single integration of the acceleration data in S1 Fig. A.

Inspection of collected raw data revealed hereby a low signal-to-noise relationship. Single integration of the acceleration data, despite the underlying drift, facilitates the extraction of mechano-biological descriptors. Hence, algorithmic determination of the number of repetitions, single repetition TUT, contraction-specific TUT and total TUT was performed by using the velocity domain.

We used a multi-analytical, algorithmic approach to obtain the mechano-biological descriptors mentioned above. First, the data was preprocessed by reducing the number of dimensions (see preprocessing algorithm). This was achieved by the acceleration vector length calculation. This procedure allows for any arbitrary spatial orientation of the smartphone to be chosen for data collection.

Outliers were removed using a Hampel filter ,which detects and removes outliers by estimating the standard deviation of each point and its neighbors. Measurement points with non-unique timestamps were excluded in order to achieve monotony of the timeseries. The timeseries was made equidistant by interpolation. The interpolation estimates the unknown function values between known data points assuming a linear relation. The offset caused by gravity was compensated for by subtracting the mean of the first and last measurement point of the timeseries.

As positive velocity values correspond with vertical weight stack raising movement and vice versa, peaks in the velocity domain reflect single repetitions. We used peak detection in the velocity domain to identify and count the number of repetitions (see repetition counting algorithm). Then, the preprocessed acceleration timeseries was subjected to a single integration. In order to reduce the effect of drift in the velocity curve, a polynomial of grade two was fitted and subtracted from the timeseries. A moving average filter was used to further smoothen the curve. Empirically, after the analysis of all 7920 performed repetitions in our real-world scenario, we used a minimum distance of 0.5 seconds between peaks and a minimum prominence threshold of one fourth of the maximum velocity magnitude as criteria for peak detection as this threshold proved to be robust.

Second, we used the result obtained from the previous algorithms to calculate the contraction-phase lengths of each repetition by finding the zero crossing points in the velocity timeseries. Due to the fact that each repetition consisted of a concentric and an eccentric phase, three zero crossing points determined the specific contraction phases as depicted in the duration calculation algorithm.
