# Supplementary figures and images for: Using smartphone accelerometer data to obtain scientific mechanical-biological descriptors of resistance exercise training
Source: PLoS One. 2020 Jul 15;15(7):e0235156. doi: 10.1371/journal.pone.0235156 (PMC7363108; doi:10.1371/journal.pone.0235156)

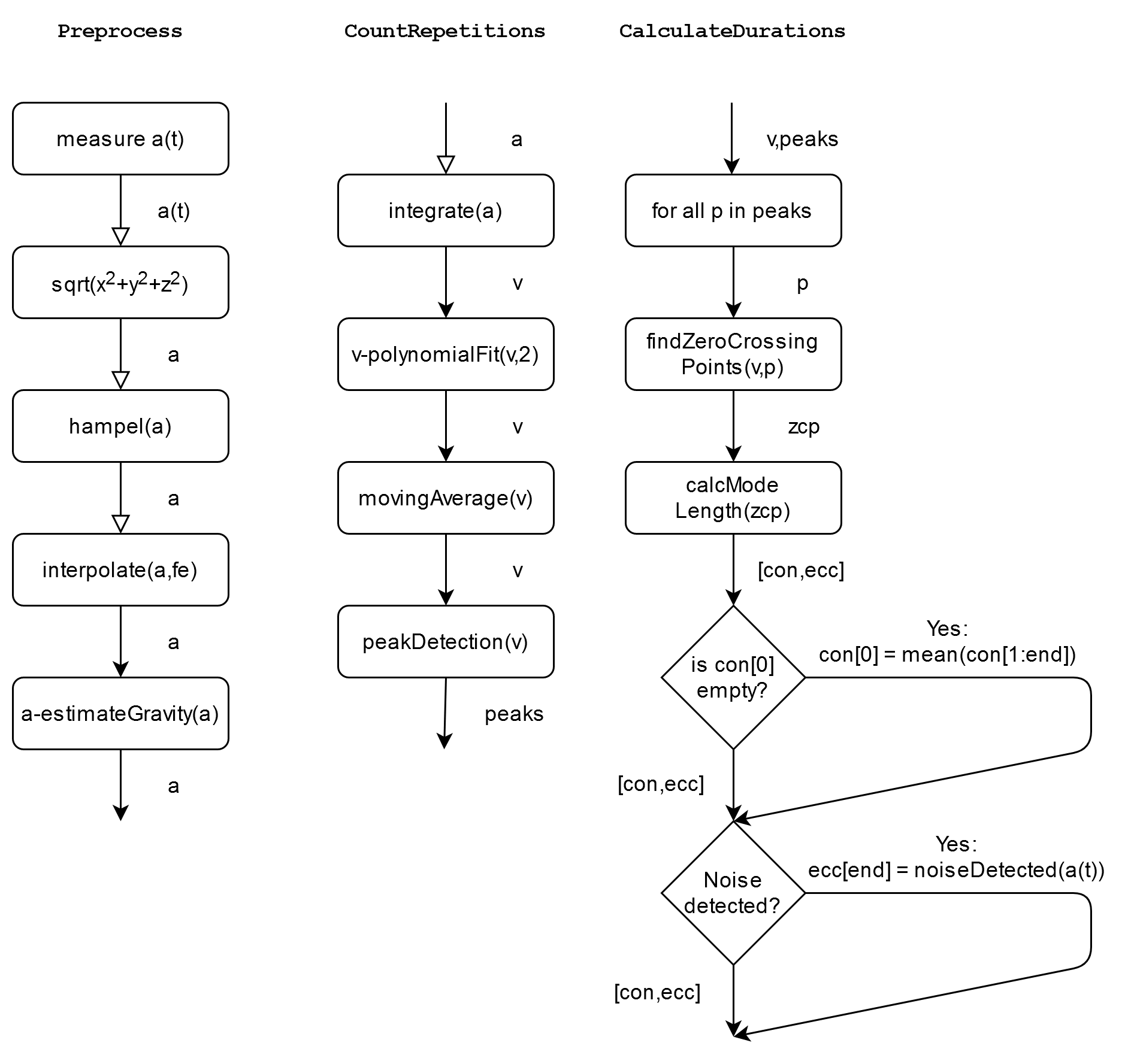

Supplement: S2 Fig — Notation: a(t) = acceleration; v(t) = velocity; fe = equidistant frequency; R = number of repetitions; ppp = position of positive peaks; zcp = zero crossing positions; pl = phase lengths. (TIF) [file pone.0235156.s002.tif]
