# Supplementary material for: Using smartphone accelerometer data to obtain scientific mechanical-biological descriptors of resistance exercise training
Source: PLoS One. 2020 Jul 15;15(7):e0235156. doi: 10.1371/journal.pone.0235156 (PMC7363108; doi:10.1371/journal.pone.0235156)
Supplement: S1 Code snippet — (DOCX) [file pone.0235156.s003.docx]

## Pseudo-code code for single repetition detection and contraction-specific phase extraction
